# Neuroprotective effects of vinpocetine, as a phosphodiesterase 1 inhibitor, on long-term potentiation in a rat model of Alzheimer’s disease

**DOI:** 10.1186/s12868-023-00790-8

**Published:** 2023-03-16

**Authors:** Meysam Shekarian, Iraj Salehi, Safoura Raoufi, Masoumeh Asadbegi, Masoumeh Kourosh-Arami, Alireza Komaki

**Affiliations:** 1 Department of Physiology, School of Medicine, Hamadan University of Medical Sciences, Shahid Fahmideh Street, Hamadan, 65178/518 Iran; 2 Department of Neuroscience, School of Science and Advanced Technologies in Medicine, Hamadan University of Medical Sciences, Hamadan, Iran; 3 Department of Neuroscience, School of Advanced Technologies in Medicine, Iran University of Medical Sciences, Tehran, Iran

**Keywords:** Alzheimer’s disease, Beta-amyloid, Vinpocetine, Phosphodiesterase1 inhibitor, Hippocampus, Long-term potentiation

## Abstract

**Background:**

Vinpocetine (Vin) is known as a phosphodiesterase 1 inhibitor (PDE1-I) drug with multilateral effects, including antioxidant and anti-inflammatory activity. In this research, we investigated the neuroprotective and therapeutic effects of Vin through hippocampal synaptic plasticity on a rat’s model of Alzheimer’s disease (AD) induced by an intracerebroventricular (ICV) injection of beta-amyloid (Aβ).

**Methods:**

Sixty adult male Wistar rats were randomly divided into six groups: 1. control, 2. sham, 3. Aβ, 4. pretreatment (Vin + Aβ): Vin (4 mg/kg, gavage) for 30 days and then, inducing an AD model by an ICV injection of Aβ(1–42), 5. treatment (Aβ + Vin): inducing an AD model and then receiving Vin for 30 days by gavage, and 7. pretreatment + treatment (Vin + Aβ + Vin): receiving Vin by gavage for 30 days before and 30 days after the induction of an AD model. After these procedures, via stereotaxic surgery, the stimulating electrodes were placed at the perforant pathway (PP) and the recording electrodes were implanted in the dentate gyrus.

**Results:**

Excitatory postsynaptic potential (EPSP) slope and population spike (PS) amplitude in the Aβ group meaningfully diminished compared to the control group after the induction of long-term potentiation (LTP).

**Conclusions:**

Vin could significantly prevent the Aβ effects on LTP. It can be concluded that pretreatment and treatment with Vin can be neuroprotective against harmful consequences of Aβ on hippocampal synaptic plasticity.

## Background

The most prevalent form of dementia is Alzheimer’s disease (AD) [1]. AD is a progressive neurodegenerative disease that occurs slowly and causes severe impairment in memory and cognitive functions, personality changes, abnormal behavior, and deterioration in thinking abilities [2]. The brains of patients with AD are found with cerebrovascular pathology and this can worsen cognitive functions in these patients [3]. Long-term potentiation (LTP) as a physiological solidarity mutual relation of synaptic plasticity that is proved to underlie memory and learning, is affected by various second messenger systems and is significantly inhibited by beta-amyloid (Aβ) in AD [4]. AD is associated with three main structural and pathological characteristics in the brain: extraneuronal aggregation of Aβ protein named Aβ plaques, intraneuronal accumulation of hyperphosphorylated tau protein named tau tangles (NFT), and finally, synaptic dysfunction and diffuse loss of neurons [5–7].

There is an increase in oxidative stress by aging and it is caused due to an imbalance in the redox state, leading to the production of excess reactive oxygen species (ROS) or the impairment of the antioxidant system [8]. Oxidative stress causes neurodegenerative disorders [9] and the brains of AD patients have a remarkable extent of oxidative damage due to the abnormal significant accumulation of Aβ and the deposition of NFT [10]. In the AD brain tissue, mitochondrial dysfunction can lead to the release of oxidative free radicals and oxidative damage. Oxidative stress markers can even be seen earlier than pathological changes in AD, and it seems that Aβ peptide is the main factor in the formation of these markers. Also, the activation of microglia by the Aβ peptide produces a high level of nitric oxide radicals [11, 12]. In addition, chronic exposure of astrocytes and microglia with Aβ peptides in the AD brain leads to the release of chemokines and some cytokines that promote inflammation and apoptosis [13–15].

Due to multifarious pathological injuries in AD, using a multi-agent drug is important [16]. Vinpocetine (Vin) is a phosphodiesterase 1 inhibitor (PDE1-I) [17–19]. Vin (a synthetic ethyl ester of the alkaloid apovincamine) is known as a PDE1-I drug with anti-inflammatory and antioxidant activity, which improves cerebral blood flow [20] and enhances memory and cognitive performance, which augments cyclic nucleotide signaling. It has also a neuroprotective effect and is effective in neurodegenerative disorders, like AD [21]. The protective effects of Vin against ROS attacks have been shown in in vitro models of oxidative stress [22]. Vin has anti-inflammatory effects through direct inhibition of the IκB kinase complex (IKK) [23]. Moreover, several studies have demonstrated the improvement in cerebrovascular flow by Vin in patients with different cerebrovascular diseases [24].

Phosphodiesterases comprise a group of enzymes that break phosphodiester bonds and hydrolyze cyclic nucleotides, and consequently, play the main role in regulating intracellular levels of the second messenger, cyclic adenosine monophosphate (cAMP), and cyclic guanosine monophosphate (cGMP) [25–27]. Two main intracellular pathways have a crucial role in synaptic and structural plasticity based on cAMP and cGMP activity. In the cAMP/PKA/CREB pathway, the cAMP-dependent protein kinase (PKA) or cAMP response element-binding protein (CREB) is activated by cAMP, which induces protein phosphorylation or gene expression, and the NO/sGC/cGMP/cGK pathway modulates long-term changes in synaptic activity and participates in different forms of memory and learning [28–30]. Due to the effect of cAMP and cGMP on neuroplasticity, a phosphodiesterase inhibitor is a potential tool for the treatment of neurological diseases [31, 32].

The most important type of synaptic plasticity that has been studied in the brain is LTP. It is a long-lasting augmentation of synaptic potency, which follows certain types of tetanic electrical stimulation, and is commonly assessed in the hippocampus and accepted as a primary mechanism of memory [33, 34]. Regarding the effect of cAMP and cGMP on neuronal plasticity and the LTP process, PDE1 inhibitors are potential factors to improve neurological disorders and increase LTP [31, 32]. The principal aim of this study was to inquire and analyze whether pretreatment and treatment with Vin can prevent AD-induced synaptic plasticity impairment in the hippocampus by evaluating the amount of LTP in the dentate gyrus (DG) of rats.

## Methods

### Animals

In this experimental study, 60 male Wistar rats from the Animal House of Hamadan University of Medical Sciences, Hamadan, Iran (weight 230 ± 15 g) were used. The rats were kept in standard conditions under a 12-h cycle of light/dark (lights from 7:00 to 19:00 h) at 22–25 °C with a humidity of 50–60%. Rats were housed in Plexiglas cages (two rats per cage). Animals had enough water and food (dry pellets of rodents) and were transferred to the animal storage room for at least ten days before the study. The protocols of animal surveillance and the procedures for treatment were according to the Veterinary Ethics Committee of the Hamadan University of Medical Science following the instructions of the National Institutes of Health on the rules of in vitro animal surveillance (NIH Publication 80–23, 1996).

### Experimental design

**Fig. 1:**
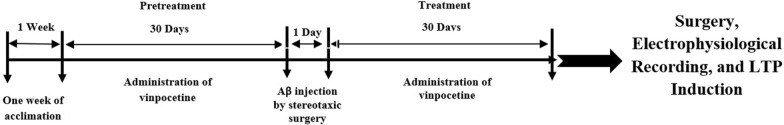
The experimental timeline. To create a rat model of Alzheimer’s disease, the rats were anesthetized with xylazine (10 mg/kg) and ketamine (100 mg/kg) 30 days after vinpocetine administration (Vin pretreatment, 4 mg/kg) in experimental groups and transferred to a stereotaxic device. The intraventricular injection of amyloid-beta (Aβ) solution (2 μL) was done at a rate of 1 μL/2 min. Following recovery, vinpocetine was re-administered through oral gavage once a day for 30 days (Vin treatment). Vin-treated rats were divided into three groups: 1. pretreatment.2. treatment. 3. pretreatment + /treatment. After treatments, in vivo electrophysiological recordings were done for the determination of the excitatory postsynaptic potential (EPSP) slope and population spike (PS) amplitude in the dentate gyrus of the hippocampus. LTP was induced through a high-frequency stimulation of the perforant pathway. For the histological study, the animals were perfused with formol-saline

After adaptation of the rats to the environment, they were randomly divided into the following groups (*n* = 10): Group 1: control, without any surgery (intact animals); Group 2: sham-operated rats that received phosphate-buffered saline (PBS) as the solvent of Aβ (1–42) via intracerebroventricular (ICV) injection; Group 3: Aβ model (AD group) rats that received single lateral ventricle injections of Aβ (1–42); Group 4: pretreatment group (Vin + Aβ) that received oral administration of Vin (4 mg/kg) for 30 days before AD induction; Group 5: treatment group (Aβ + Vin) that received the oral administration of Vin (4 mg/kg) after AD induction for 30 days; and Group 6: pretreatment + treatment group (Vin + Aβ + Vin) that received Vin (4 mg/kg) 30 days before and 30 days after AD induction (Fig. 1).

### Main reagents and drugs

In order to prepare amyloid fibrils, as a neurotoxic factor, based on the instruction, 100 μg lyophilized powder Aβ (1– 42) (Tocris Bioscience; Bristol, UK) was dissolved in 100 μL of PBS as a solvent, followed by incubation at 37 °C for seven days before use [35].

### The dose of vinpocetine and duration of treatment

The dose of Vin in our study was selected based on previous research [36–38]. Thus, in this investigation, Vin was administrated orally (gavage) once a day at 8:00 a.m. at a dosage of 4 mg/kg for 30 sequential days. For the pretreatment + treatment group (Vin + Aβ + Vin), Vin was gavaged 30 days before and 30 days after Aβ induction.

### Aβ injections and surgery

Animals were anesthetized using a combination of xylazine (10 mg/kg) and ketamine (100 mg/kg) and then, placed in the stereotaxic device (Stoelting Co., Wood Dale, IL, USA). The stereotaxic rods were placed inside the animal's ears and after observing the eyes reflex, the head was fixed in the device. Then, the bregma and lambda regions were found and according to the Paxinos and Watson rat brain atlas, the coordinates of the brain ventricular regions were adjusted and one tiny hole was fixed in the right ventricle. Also, 5 μl of Aβ(1–42) was unilaterally injected by a 5 μl microsyringe(Hamilton Laboratory Products, Reno, NV, USA) through its stainless steel cannula in the right lateral ventricle using the coordinates of the dorsal/ventral: 4.0 mm, medial/lateral: 1.4 mm, and anteroposterior: -0.8 mm from bregma. Injections lasted 6 min and the needle of the microsyringe remained in the hole for 3 min after the injection to make sure that the injection of Aβ(1–42) is completely done [39]. Instead of the Aβ(1–42), the same amount of PBS was injected into the rats' cerebroventricular in the sham group. After injection, the scalp was sutured and the rats were transported to their cages. It takes two weeks to create an AD model [40].

### Surgical procedures, electrophysiological recordings, and LTP induction

**Fig. 2:**
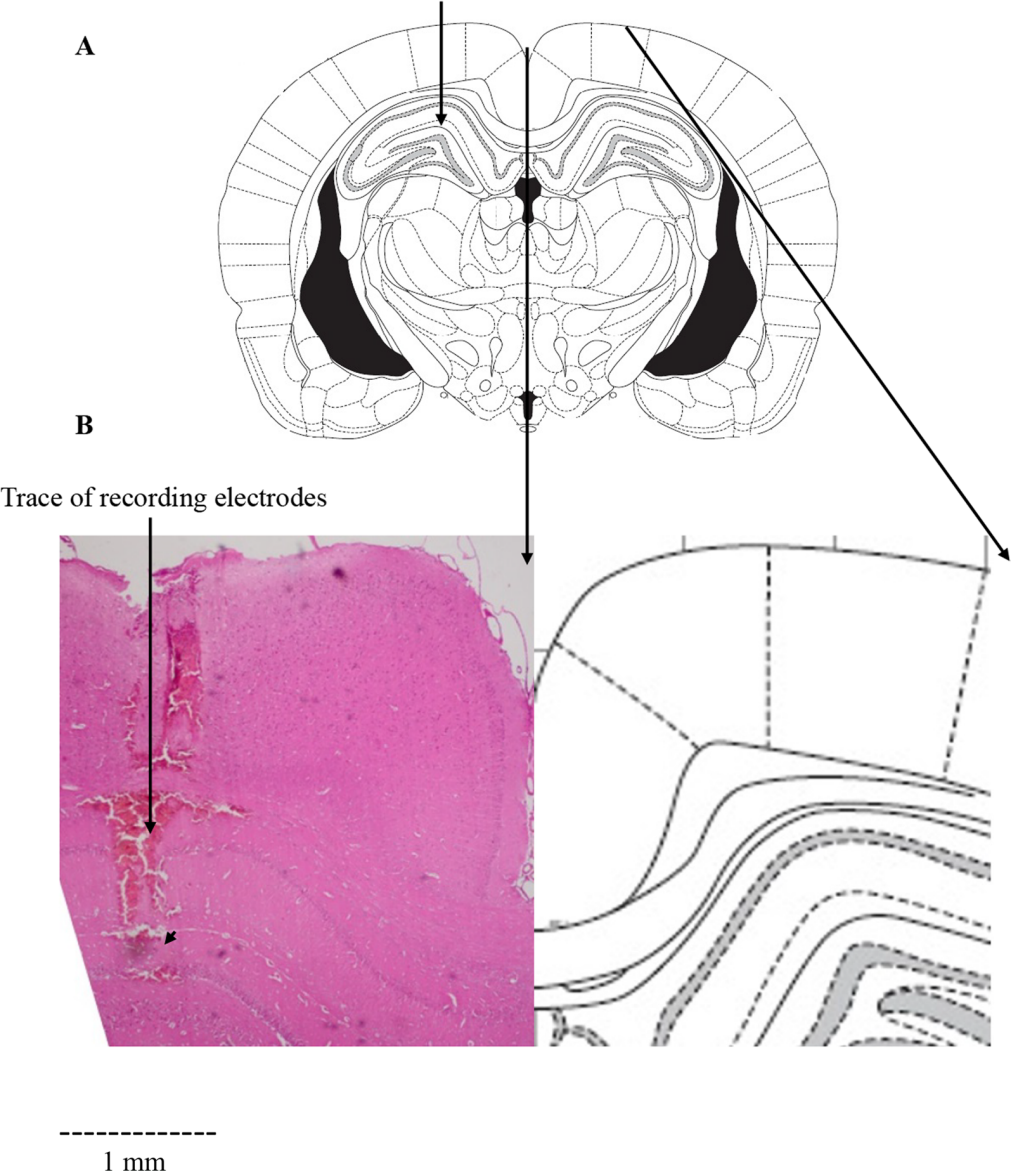
Schematic drawing of a rat brain coronal section from Paxinos and Watson, showing the trace of recording electrodes (arrow) in the dentate gyrus (DG) (**A**). The cross-section view of the hippocampal area with the tip of recording electrodes (arrowhead) in DG; sample on left and atlas plate on right (**B**). Scale bar: 1 mm

Initially, the rat was anesthetized by an intraperitoneal injection of urethane (1.5 g / kg) [41, 42]. Then, the animal was placed in the stereotaxic device, and using a heating pad, the animal's body temperature was maintained in the natural range (37.0 ± 0.2 °C). After opening the skin of the skull based on the Paxinos and Watson rat brain atlas [43], the lateral perforant path (PP) and DG were determined. Based on a horizontal skull surface, the coordinates of PP were 4.3 mm lateral to the midline, 8.1 mm posterior to the bregma, and 3.2 mm ventral below the skull surface, and the coordinates of DG were 2.3 mm lateral to the midline and 3.8 mm posterior to the bregma. After creating holes in the skull, two concentric stainless-steel bipolar electrodes were placed in these points. Teflon-coated stimulating electrodes (except for the tips) 125 μm in diameter were used. The stimulating electrode was placed in PP and the recording electrode in DG. The recording electrode was moved down into the DG (usually 2.7–3.2 mm ventral) until the utmost field excitatory postsynaptic potentials (EPSPs) were observed (Fig. 2). To attain the optimal ventral placement, we monitored the electrophysiological response that was extracted from the DG following single-pulse PP stimulation. To minimize trauma to the brain tissue, the electrodes from the cortex to the hippocampus were entered very slowly (0.2 mm/min). By stimulating the PP to specify the stimulus intensity to be utilized in each rat (40% maximum population spike (PS)), input–output current profiles were obtained. Through constant current isolation units at a frequency of 0.1 Hz, single biphasic square wave pulses (0.1 ms) were delivered. Following stimulation of the PP, the field potential responses were obtained in the granular cells of the DG.

Every 10 s, the test stimuli to the PP were applied. The electrodes were placed to extract the utmost field EPSPs (fEPSP) and PS amplitudes. LTP with high-frequency stimulation (HFS) protocol (0.2-ms stimulus duration, 10 bursts of 20 stimuli, 10-s interburst interval, 400 Hz) was induced after making sure of a response of constant-state baseline, which ordinarily takes nearly 50 min. LTP induction was done at a stimulus intensity that evoked PS amplitudes and fEPSP slopes that were almost 80% of the maximum response. To determine any changes in the synaptic responses of DG neurons, both fEPSPs and PSs were recorded 5, 30, and 60 min after the HFS. For each time point, an average of ten responses was continuously evoked at 10-s stimulus intervals [41, 42, 44–49].

The parameters of the stimulations were determined with relevant software. Then, a constant current isolator unit(A365, World Precision Instruments, Inc.) was set via the derived data prior to transferring it to the PP. The DG's field potential responses were passed through a preamplifier(Differential amplifier DAM 80, World Precision Instruments, Inc. Sarasota, FL, USA), and amplified 1000 times while they were filtered(bandpass, 1 Hz to 3 kHz). These responses were digitized at a sampling rate of 10 kHz, which were visible on a monitor and oscilloscope.

### Measurement of evoked potentials

**Fig. 3:**
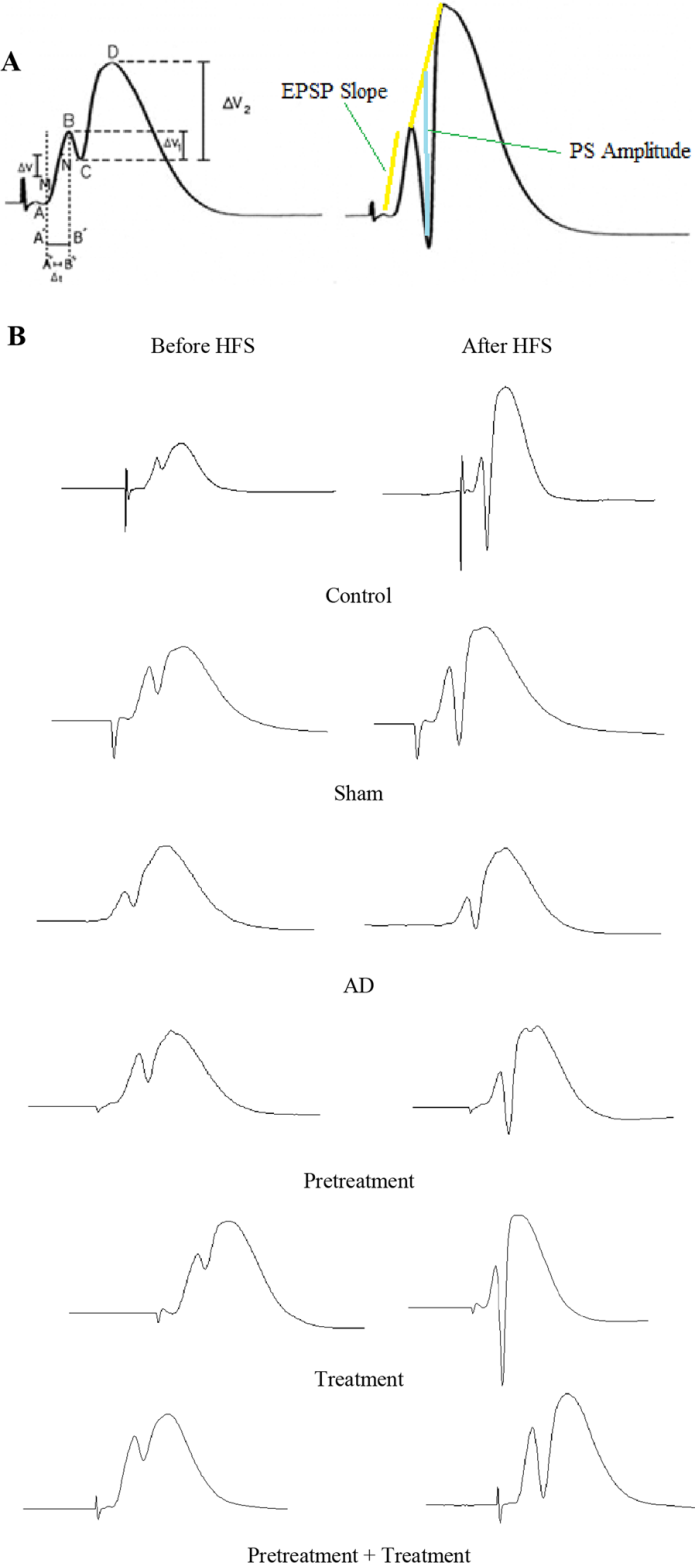
Population spike (PS) amplitude and field excitatory postsynaptic potential (fEPSP) slope, assessed in a representative sample field potential in the hippocampus of the control rats (**A**). Sample traces of evoked field potential were recorded in the dentate gyrus (DG) of the hippocampus before and following high-frequency stimulation (HFS) of the perforant pathway (PP) in all groups (**B**)

PS and fEPSP are two components of the evoked field potential in the DG. During the electrophysiological recordings, the alterations in the PS amplitude and fEPSP slopes were evaluated. The PS amplitude is equal to the head of the first positive deflection of the evoked potential to the next negative potential head. The fEPSP slope is equal to the slope of the line linking the start of the evoked potential first positive deflection to the second positive deflection head. The fEPSP slopes were measured between 20 and 80% of the peak amplitude (Fig. 3) [41, 42, 44–47, 49]**.** The stimulation intensity was regulated to evoke potentials, which consisted of 40% of the maximum PS amplitude, determined by an input/output curve.

$$PS = \frac{{\Delta v_{1} + \Delta v_{2} }}{2} = \frac{BC(v) + CD(v)}{2}$$

$$EPSP = \frac{\Delta v}{{\Delta t}} = \frac{MN(v)}{{A^{\prime\prime}B^{\prime\prime}}}$$

$$A^{\prime\prime}B^{\prime\prime} = \frac{{A^{\prime}B^{\prime}}}{3}$$

### Data analysis

We analyzed data using repeated measure analysis of variance pursued by Tukey’s test using GraphPad Prism softwareversion 7.0. Values are represented as mean ± SEM. P-values less than 0.05 (P < 0.05) were considered significant.

The LTP value was determined using the following equation:

$${\text{LTP}} = { }\frac{{\text{The PS value or EPSP after HFS induction }}}{{\text{Average of the PS or EPSP at baseline}}} \times { 1}00\%$$

## Results

After the HFS of the PP, field potential responses were found in granular cells in the DG (Fig. 3).

### Effects of Vin pretreatment and treatment and both on the EPSP slopes of granular cells in the DG of AD rats

**Fig. 4:**
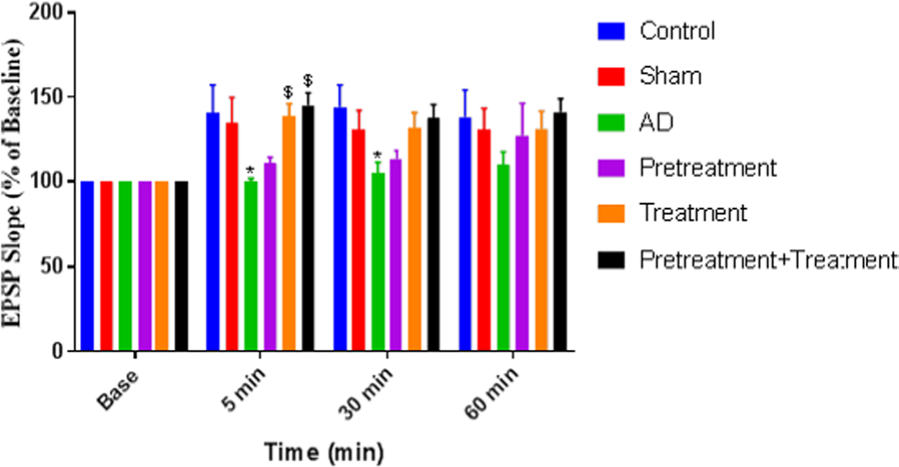
The effect of pretreatment, treatment, and pretreatment + treatment with Vinpocetine (Vin) on excitatory postsynaptic potential (EPSP) slope in the dentate gyrus (DG) utilizing 400 Hz tetanization of the AD-induced rats. Long-term potentiation (LTP) of the EPSP slope in DG granular cell synapses is meaningfully dissimilar between groups. Values are represented as the mean ± SEM% of the baseline. *: P < 0.05 compared to the control group and $: P < 0.05 compared to the AD group

**Fig. 5:**
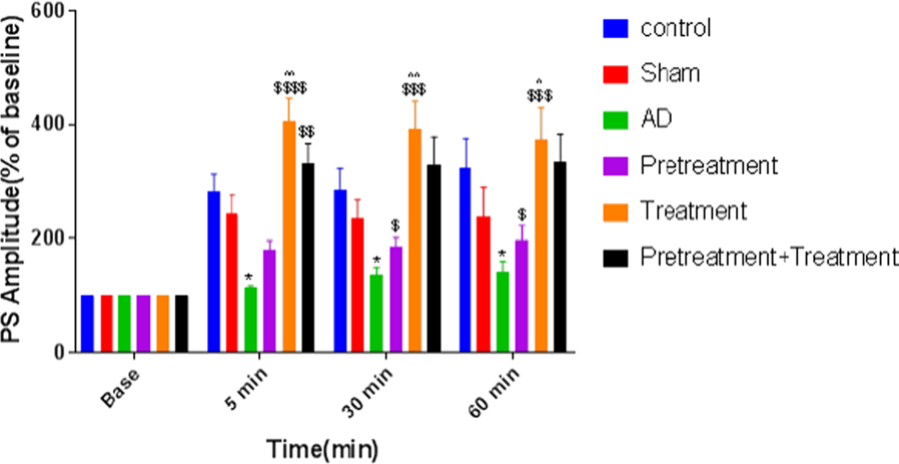
Effect of pretreatment, treatment, and pretreatment + treatment with Vinpocetine (Vin) on values of population spike (PS) in the dentate gyrus (DG) utilizing 400 Hz tetanization. Long-term potentiation (LTP) of PS in DG granular cells in the hippocampus is meaningfully dissimilar between groups. Values are represented as the mean ± SEM% of the baseline. *: P < 0.05 compared to the control group; $: P < 0.05, $$: P < 0.01, $$$: P < 0.001, and $$$$: P < 0.0001 compared to the AD group; and ^: P < 0.05 and ^^: P < 0.01 compared to the pretreatment + treatment group

We induced LTP in the DG by HFS of the PP. The effects of Vin pretreatment and treatment (prior to and after Aβ injections) and both on the LTP of the PS amplitude and the EPSP slopes of the AD rats are shown in Figs. 4 and 5, respectively.

The EPSP slope was 134.75 ± 16.12% in the control rats. After HFS, there was a significant decrease (P < 0.05) in the EPSP slope of the AD group (105.25 ± 2.16%; n = 8) compared to the control and sham groups (119.75 ± 15.39%). There was also a significant increase (P < 0.05) between the treatment (138.5 ± 11.30%) and pretreatment + treatment groups (136.12 ± 9.67%) compared to the AD group. However, no significant difference was found between the pretreatment (113.12 ± 4.12%) and AD groups (Fig. 4).

### Effects of Vin pretreatment and treatment and both on the PS amplitude of granular cells in the DG of AD rats

The PS amplitude was 283.75 ± 18.24% in control rats. The range of PS amplitude of the AD group (113.5 ± 2.78%) decreased significantly (P < 0.05) compared to the control group. Administration of Vin significantly increased the PS amplitude in the treatment (408 ± 22.98%; P < 0.0001)) and pretreatment + treatment (331 ± 19.45%; P < 0.01) groups compared to the AD group. However, there was no significant difference between the pretreatment (181 ± 13.39%) and AD groups. There was a significant difference (P < 0.01) between the treatment and pretreatment groups in terms of PS amplitude (Fig. 5).

## Discussion

We evaluated the neuroprotective effects of Vin on the AD model induced by ICV Aβ injection in rats using an LTP assay. In the current study, ICV injection of Aβ was used to induce AD. Aβ inhibited LTP in the DG by reducing both the EPSP slope and PS amplitude in the AD group compared to the control group. In many studies, the LTP process was inhibited in AD models and a significant reduction was observed in the EPSP slope and PS amplitude after HFS [50–53].

In the present study, we evaluated the LTP in the hippocampus of AD rats. The hippocampus is considered a classic model to study synaptic plasticity, such as LTP and LTD [54]. Hippocampal LTP is a model of synaptic plasticity with a direct association with memory and learning and is repressed after exposure to Aβ [4, 55, 56]. The hippocampus is known as one of the first areas of the brain that is affected in the memory process and in AD [7].

Our results indicated that Vin administration in the pretreatment, treatment, and pretreatment + treatment groups improved LTP in granular cells in the DG by increasing the EPSP slope and the PS amplitude in comparison with the AD group. It has been shown that Vin facilitates LTP [57], increases the dynamics of dendritic spines [58], improves memory retrieval in passive avoidance tasks in rats [59], and boosts cognitive efficiency in humans [19].

Increasing the levels of intracellular cGMP and cAMP via the phosphodiesterase 1 inhibitory effect of Vin leads to the phosphorylation of AMPA receptors and their incorporation and attachment to the synapses [60]. Long-term phosphorylation of AMPA receptors is involved in the LTP of hippocampal synapses [61]. Cyclic nucleotide plays a significant role in cognitive function and the levels of cyclic nucleotides, especially cAMP and cGMP are changed in AD [62, 63]. Thus, it is obvious that in the present study, the cyclic nucleotide restoration by Vin may act as an effective strategy to ameliorate cognitive functions and synaptic plasticity in AD.

Another explanation for the effect of Vin on LTP in the current study might be its antioxidant activity. Studies on AD have shown oxidative stress production and severe oxidative damage associated with two pathological characteristics of AD, Aβ, and NFT destructions [64, 65]. Vin has antioxidant activity by eliminating hydroxyl radicals and acts as an antioxidant by preventing the production of ROS and lipid peroxidation in brain synaptosomes [66, 67]. In this regard, Vin remarkably reduces the oxidative–nitrite stress by a decrease in malondialdehyde (MDA) and nitrite levels and restituting a decrease in glutathione (GSH) levels [21, 37]. Furthermore, Vin has antioxidant activity and prevents reactive free radical generation, which plays a role in a decrease in high glucose-induced oxidative damage [68]. Also, in another recent experiment, Vin improved memory and learning impairment after Aβ injection because of its antioxidant effects. Therefore, Vin is capable of changing the balance between oxidants and antioxidants, in favor of antioxidants to cause an improvement in LTP reduction induced by Aβ.

Another description for the improvement of LTP by Vin in AD rats in the existing study might be its neuroprotective effect. Vin possesses a neuroprotective effect because of its anti-inflammatory activities [69] through the AMPK signal pathway phosphorylation [70] and the nuclear factor κB (NF-κB) pathway [71, 72] to inhibit the expression of inflammatory genes [73]. Furthermore, Vin inhibits the release of TNF-α-stimulated inflammatory agents by inhibiting the IκB kinase complex (IKK)/ NF-κB pathway [74]. Therefore, because Vin exerts an anti-inflammatory role and can improve cognitive properties, it can be considered as an option for the treatment of neurodegenerative diseases, like AD.

In the current study, we evaluated the PS amplitude and EPSP slope 5, 30, and 60 min after HFS to find how long the effect of Vin on LTP lasts. Vin treatment and pretreatment + treatment, potentiated the reduction in EPSP slope and PS amplitude by Aβ injection 5 min after HFS. Therefore, Vin treatment and pretreatment/treatment can be efficient in the improvement of Aβ-induced diminution of LTP just for short time after HFS. In addition, Vin pretreatment and also its treatment could improve the reduction of PS amplitude by Aβ injection 30 and 60 min after HFS. Therefore, Vin treatment can improve the Aβ-induced diminution of PS amplitude in all the time points after HFS in the current study. The increment of PS amplitude both 30 and 60 min after applying HFS might display the long-lasting enhancement of LTP by Vin treatment. Therefore, it seems that the Vin treatment might enhance the LTP for a longer time due to its effect on long-lasting processes, such as second messenger systems and protein synthesis. Changes in long-lasting processes, such as the production of the second messengers via the PDE1 inhibitory effect of Vin can indicate the longer effects of Vin on LTP. The cAMP second messenger activates cAMP response element-binding protein (CREB) signaling through the protein kinase A (PKA) leading to adjusting the transcription of synaptic plasticity genes, like the brain-derived neurotrophic factor (BDNF) protein gene [75–86]. CREB also can lead to the expression of several neuroprotective and anti-apoptotic molecules, including fibroblast growth factor (FGF) and transforming growth factor (TGF) as the protective neurotrophic factors [87], Bcl-2 as an anti-apoptotic protein [88], and peroxisome proliferator-activated receptor-gamma coactivator 1 alpha (PGC-1α) as a ROS scavenger [89].

In this study, we used oral administration of Vin (4 mg/kg). In a previous study by Molnár et al., on the LTP of DG, Vin at 0.1 and 5 mg/kg failed to increase the amplitude of PS [57]. It seems that Vin at a dose of 4 mg/kg can induce the potentiation of LTP reduction by Aβ. Therefore, Vin at a dose of 4 mg/kg might show therapeutic effects in ameliorating the LTP reduction induced by Aβ.

## Conclusion

In summary, our results suggest that Vin can improve the Aβ-induced impairment of neuronal plasticity. We also suggest that pretreatment and treatment with Vin, according to its neuroprotective, antioxidant, anti-inflammatory, and multi-functional effects, can prevent Aβ-induced impairment in synaptic plasticity in the hippocampal PP-DG pathway evidenced by the evaluation of LTP. Therefore, Vin has a preventive and therapeutic effect on AD. However, more studies are needed to assess the effectiveness of Vin in AD in humans.

## Data Availability

All data generated or analysed during this study are included in this published article [and its supplementary information files].
